# Supplementary material for: Packaged Foods Labeled as Organic Have a More Healthful Profile Than Their Conventional Counterparts, According to Analysis of Products Sold in the U.S. in 2019–2020
Source: Nutrients. 2021 Aug 29;13(9):3020. doi: 10.3390/nu13093020 (PMC8469099; doi:10.3390/nu13093020)
Supplement: Supplementary file 1 [file nutrients-13-03020-s001.zip › nutrients-1328023-supplementary.pdf]

nutrients MDPI

Supplementary Table

Table S1. Ingredients classified as ultra-processed and their cosmetic additive status. Functional groups of ingredients that are classified as cosmetic additives are marked with an asterisk *.

Ultra-Processed Ingredient Function	Ingredient
**Anti-foaming***	Dimethyl Polysiloxane
	Fatty Acids
	Fumed Silica
	Silica Gel
	Silicon Dioxide
**Anticaking Agent**	Aluminum Silicate
	Calcium Aluminosilicate
	Calcium Silicate
	Powdered Cellulose (To Prevent Caking)
	Rice Concentrate
	Sodium Aluminosilicate
	Sodium Ferrocyanide
	Talc Or Magnesium Silicate (553, 553B)
**Bulking Agent***	Cellulose & Associated Derivatives
	Chicory Root & Associated Derivatives
	Cottonseed Fiber
	Fructan
	Galactooligosaccharides (Gos) (A Type of Prebiotic)
	Hydroxypropyl Methylcellulose
	Inulin
	Isomalto-Oligosaccharides
	Methylcellulose
	Oligofructose
	Oligosaccharides
	Short-Chain Fructooligosaccharides
	Sodium Carboxymethylcellulose
	Soy Bran
	Soybean Fiber
	Xylo-Oligosaccharides
**Color Stabilizer**	Aluminum Ammonium Sulfate
	Calcium Disodium EDTA
	Carbon Monoxide
	Citric Acid
	Disodium Dihydrogen Pyrophosphate (To Retain Color)
	Disodium EDTA (To Promote Color Retention)

	EDTA<br>Erythorbic Acid (To Promote Color Retention)<br>Ethoxyquin To Preserve Color<br>Ferrous Gluconate (To Stabilize Color)<br>Filtered Wood Smoke (To Promote Color Retention)<br>Magnesium Carbonates<br>Magnesium Hydroxide<br>Malic Acid (To Promote Color)<br>Potassium Metabisulfite (To Protect Color)<br>Potassium Nitrate<br>Sodium Nitrate<br>Sodium Nitrite<br>Sodium Sulfate<br>Tasteless Smoke<br>Zinc Chloride (Color Stabilization)
**Color***	Alkalized Cocoa (Color)<br>Annatto & Annatto Derivatives<br>Anthocyanin<br>Apo Carotenal (Color)<br>Apple & Associated Derivatives (Color)<br>Artificial Colors (All Varieties)<br>Astaxanthin<br>Beet & Associated Derivatives (Color)<br>Berry Color<br>Beta-Carotene<br>Blackcurrant & Associated Derivatives (Color)<br>Blue 1 & Blue 1 Lake<br>Blue 2 Lake<br>Blueberry & Associated Derivatives (Color)<br>Cabbage & Associated Derivatives (Color)<br>Canthaxanthin<br>Caramel & Associated Derivatives (Color)<br>Carmine (Color)<br>Carotenes (160, 160A, 160E, 160F)<br>Carrot & Associated Derivatives (Color)<br>Cherry Juice & Concentrate (Color)<br>Chlorophyll And Chlorophyllins<br>Chlorophyll Copper Complex<br>Chokeberry Juice & Concentrate (Color)<br>Cochineal, Carmine, And Carminic Acid<br>Color<br>Colour (E150C)

Copper Chlorophyllin (Color)
Cranberry Powder (Color)
Egg Shade
Elderberry & Associated Derivatives (Color)
FD&C Blue #2
FD&C Green #3
FD&C Red
FD&C Red #3 Lake
FD&C Red #4
FD&C Red #40 Aluminum Lake
FD&C Red No. 4 Lake
Ferrous Gluconate
Fruit & Vegetable Derivatives (Color)
Grape & Associated Derivatives (Color)
Hibiscus Derivatives (Color)
Huito & Huito Juice Concentrate (Color)
Iron Oxide
Lycopene
Malt
Mica
Natural Colors (All Varieties)
Oleoresin Capsicum
Orange 4
Paprika & Associated Derivatives (Color)
Plum Juice Concentrate (Color)
Pomegranate Extract
Pumpkin Derivatives (Color)
Purple Sweet Potato & Associated Derivatives (Color)
Radish & Associated Derivatives (Color)
Red 3 Lake
Red 40
Red 43 Lake
Red Bell Pepper Powder (Color)
Red Cabbage & Associated Derivatives (Color)
Red Currant Powder (Color)
Riboflavin (Color)
Rice Starch (Color)
Saffron (Color)
Spice And Color
Spinach Derivatives (Color)
Spirulina & Associated Derivatives (Color)
Suma Root

	Titanium Dioxide (Color)
	Tomato & Associated Derivatives (Color)
	Turmeric & Associated Derivatives (Color)
	Vegetable & Associated Derivatives (Color)
	Vitamin A Palmitate (Color)
	Watermelon Juice Concentrate (Color)
	Yellow #5 & #6 And #5 &#6 Lake
	Zinc Oxide
**Emulsifier***	Acetic & Fatty Acid Esters of Glycerol (472, 472A)
	Ammonium Salts of Phosphatidic Acid
	Calcium Stearoyl Lactylate
	Citrus Pulp (Dried)
	Cyclodextrin
	Diacetyltartaric & Fatty Acid Esters of Glycerol (472E)
	Diglycerides
	Disodium Orthophosphate (339(II))
	Disodium Phosphate
	Esters Of Fatty Acids
	Glycerin Or Glycerol
	Glycerol Esters of Wood Rosins
	Glycerol Monostearate
	Glyceryl Monostearate
	Lactic & Fatty Acid Esters of Glycerol (472B)
	Lactic Acid Esters of Mono and Diglycerides of Fatty Acids
	Lecithin
	Mono And Diglycerides of Fatty Acids
	Monoglycerides
	Oleyl Lactylic Acid
	Orange Pulp (Dried)
	Plant Mono and Diglycerides
	Polyethylene Glycol
	Polyglycerol Esters of Fatty Acids
	Polyglycerol Polyricinoleate
	Polyoxyethylene Mono and Diglycerides of Fatty Acids
	Polysorbate 20, 60, 65, and 80
	Propylene Glycol Esters of Fatty Acids
	Propylene Glycol Mono and Diesters of Fatty Acids
	Propylene Glycol Monoester
	Propylene Glycol Monostearate
	Rapeseed
	Sodium Caseinate
	Sodium Lauryl Sulfate

	Sodium Stearoyl Lactylate
	Sorbitan Monolaurate
	Sorbitan Monostearate
	Sorbitan Tristearate
	Stearoyl Lactylate
	Steroyl Lactylic Acid (Sla)
	Sucrose Acetate Isobutyrate
	Sucrose Esters of Fatty Acids
	Sunflower Lecithin
	Tetrapotassium Diphosphate (450(V))
	Triethyl Citrate
**Fermented and Distilled Alcohol**	Alcohol
	Amaretto
	Bourbon
	Brandy
	Cognac
	Frangelico
	Gin
	Irish Cream Liqueur
	Liqueur
	Liquor
	Port
	Rum
	Tequila
	Vodka
	Whiskey
**Firming Agent**	Alum
	Aluminum Potassium Sulfate
	Aluminum Sulfate
	Calcium Hydroxide
	Magnesium Chloride
**Flavor Enhancer***	Amino Acids
	Dipotassium Guanylate
	Disodium 5'-Ribonucleotides
	Disodium Guanosine 5'-Monophosphate
	Disodium Guanylate
	Disodium Inosinate
	Erythorbic Acid
	Glutamates
	Glutamic Acid
	Glycine
	Hydrolyzed Proteins (All Varieties)

	Hydrolyzed Wheat
	Inosinic Acid
	L-Glutamic Acid
	Magnesium Sulfate
	Monosodium Glutamate (MSG)
	Nucleotides
	Potassium Sulfates
	Ribonucleotides
	Sodium Ribonucleotides for Flavor
**Flavor***	Adipic Acid
	Aldehydes
	Anchovy Flavor
	Apple Flavor
	Aqueous Natural Smoke
	Artificial Flavors (All Varieties)
	Autolyzed Yeast Extract
	Avocado Flavor
	Bacon Flavor
	Baileys Flavoring (Milk)
	Banana Flavor
	Barbeque Flavor
	Basil Flavor
	Beef Flavor
	Benzaldehyde
	Benzyl Alcohol
	Blueberry Flavor
	Burnt Sugar Flavor
	Butter Acids
	Butter Esters
	Butter Flavor
	Buttermilk Flavor
	Butyric Acid
	Caffeine
	Cake Flavor
	Caramel Flavor
	Cassava Extract
	Chai Flavor
	Cheese Flavor
	Cherry Flavor
	Chicken Flavor
	Chicken Stock (Flavored)
	Chili Flavor

Cinnamic Aldehyde
Cinnamon Flavor
Coconut Flavor
Coffee Flavor
Cookie Dough Flavor
Cookie Flavor
Creme De Menthe Flavor
Diacetyl
Dill Flavor
Disodium Succinate
Dragon Fruit Flavor
Elderberry Flavor
Essence (All Varieties)
Ethyl Maltol
Ethylvanillin
Flavor
Formic Acid
Fragrance & Natural Fragrance
French Toast Flavor
Fried Flavor
Garlic Flavor
Ginger Flavor
Glutamine
Glyceryl Triacetate
Graham Cracker Flavor
Grape Flavor
Green Chili Flavor
Grill Flavor
Hibiscus Flavor
Honey Flavor
Hot Sauce Flavor
Hydrolyzed Corn
Hydrolyzed Corn Gluten
Imitation Almond Extract
Isopropanol
Jalapeno Flavor
Lavender Flavor
Lemon Flavor
Lime Flavor
Limonene
Liquid Smoke
Malt Flavor

Maltol
Mango Flavor
Maple Flavor
Meaty Flavor
Menthol
Milk Flavor
Mint Flavor
Mojito Flavor
Mushroom Flavor
Natural & Artificial Flavor (All Varieties)
Natural Flavor (All Varieties)
Nut Flavor
Onion Flavor
Orange Flavor
Oyster Flavor
Pandan Flavoring
Peach Flavor
Pecan Flavor
Pepper Flavor
Peppermint Flavor
Pineapple Flavor
Pistachio Flavor
Pomegranate Flavor
Pork Broth with Natural Flavorings
Pork Flavor
Potassium Acetate
Poultry Flavor
Pumpkin Spice Flavor
Quillaia Extract
Quillaja
Raspberry Flavor
Roast Flavor
Rye Flavor
Saponaria Extract
Scallop Flavor
Sherry Wine Flavor
Smoke Flavor
Sodium Acetate
Sour Cream Flavor (Non Dairy)
Soy Sauce Flavor
Spearmint Flavor
Spice Flavor

	Spinach Flavoring
	Starter Distillate
	Stearic Acid
	Strawberry Flavor
	Tannic Acid
	Tomato Flavor
	Tropical Flavors
	Truffle Flavor
	Turkey Flavor
	Vanilla Flavor
	Vanillin
	Vegan Flavoring
	Vegetarian Flavor
	Wasabi Flavor
	Wildberry Flavor
	Yeast Extract
	Yogurt Flavor
**Gelling Agent***	Gelatin
**Glazing Agent***	Beeswax, White and Yellow
	Carnauba Wax
	Confectioner's Glaze
	Confectionery Coating
	Paraffin Wax (905C)
	Plant Resin
	Shellac
	Wax
**Humectant**	Citrus Fiber
	Citrus Flour
	Humectant
	Propylene Glycol
**Rare Culinary Use**	100% Muscadine Juice Reconstituted
	Almond Protein
	Apple Juice Concentrate
	Banana Juice Concentrate
	Barley Gluten
	Barley Malt
	Barley Malt Extract & Extract Powder
	Buttermilk Powder
	Buttermilk Solids
	Calcium-Sodium Caseinate
	Casein
	Cassava Syrup

Cheese Curds
Chia Protein Powder (Salvia Hispanica)
Collagen
Corn Gluten
Corn Sugar
Corn Sweeteners
Corn Syrup
Corn Syrup Solids
Crystalline Fructose
Cultured Non-Fat Milk Solids
Cultured Whey & Associated Derivatives
Dairy Solids
Date Juice Concentrate
Date Powder
Date Sugar
Date Syrup
Dextrose
Dried Buttermilk
Dried Honey
Dried Milk (Fat Free)
Edible Collagen Casings
Emulsified Vegetable Shortening
Extract Of Malted Barley and Corn
Faba Bean Protein
Fructose
Fructose Powder
Fructose Solids
Fruit Syrup
Fully Hydrogenated Oils (All Varieties)
Gelatin Hydrolysate
Glucose - Fructose Syrup
Glucose & Associated Derivatives
Golden Syrup
Guanabana Juice
Hemp Protein & Hemp Protein Concentrate
Hi-Maltose Corn Syrup
High Fructose Corn Syrup
Honey Crystals
Honey Powder
Honey Solids
Hydrogenated Animal Fats and Oils
Hydrogenated Lard

Hydrogenated Oils (All Varieties)
Hydrogenated Palm Stearin
Hydrogenated Tallow
Hydrogenated Vegetable Fat
Hydrogenated Vegetable Shortening (Soy)
Hydrolyzed Collagen
Interesterified Oils (All Varieties)
Invert Sugar & Invert Sugar Syrup
Kiwifruit Juice Concentrate
Lactose
Malt & Associated Derivatives
Maltodextrin
Maltose
Maltose Syrup
Maltrin
Mandarin Juice Concentrate
Mango Juice Concentrate
Maqui Berry Juice Concentrate
Margarine (Baker's)
Micellar Casein
Milk (Fat Free) (Dried)
Milk Concentrate
Milk Powder
Milk Protein Concentrate
Milk Protein Isolate
Milk Solids
Milk Sugars
Mulberry Juice
Mycoprotein
Nonfat Dry Milk
Oat Extract
Oat Isolate
Oat Syrup Solids
Orange Juice Concentrate
Partially Hydrogenated Oils
Partially Hydrogenated Vegetable Fat
Passionfruit Juice Concentrate
Pea Flour
Pea Powder
Pea Protein & Pea Protein Concentrate
Pea Protein Isolate
Peach Juice Concentrate

Pear Juice Concentrate
Pineapple Concentrate Powder
Pineapple Juice Concentrate
Pineapple Syrup
Plum Juice Concentrate
Pomegranate Juice Concentrate
Potassium Caseinate
Potato Protein
Protein Isolate
Prune Juice Concentrate
Raisin Juice Concentrate
Raspberry Juice Concentrate
Rbst-Free Dry Milk
Refined, Deodorized, Partially Hydrogenated, Fractionated Palm Oil
Rennet Casein
Rice Malt
Rice Protein
Rice Syrup
Shortening & Shortening Powder
Soluble Raisin Solids
Sorghum Syrup
Soy Bean Milk Powder
Soy Concentrate
Soy Flour
Soy Grits
Soy Isolate
Soy Meal
Soy Nuggets
Soy Protein & Soy Protein Isolate
Soy Pulp
Soybean Flakes
Soybean Powder
Sprouted Rice Protein
Starch Syrup
Strawberry Juice Concentrate
Syrup
Tangerine Juice Concentrate
Tapioca Syrup
Tapioca Syrup Solids
Textured Soy Protein
Textured Vegetable Protein
Vegetable Protein

	Vegetable Shortening<br>Vegetable Shortening (Interesterified and Hydrogenated Soybean Oils)<br>Wheat Gluten<br>Wheat Isolate<br>Wheat Protein & Wheat Protein Isolate<br>Wheat Syrup<br>Whey & Associated Derivatives<br>Whole Milk Powder (Rbst Free)<br>Xylose<br>Yacon Syrup
**Sequestrant**	Tetrasodium Pyrophosphate<br>Dipotassium Acid Phosphate<br>Dipotassium Phosphate<br>Disodium Dihydrogen Pyrophosphate<br>Disodium Pyrophosphate<br>Glucono Delta-Lactone<br>Monosodium Phosphate<br>Potassium Phosphates<br>Sodium Acid Pyrophosphate<br>Sodium And Potassium Triphosphates<br>Sodium Gluconate<br>Sodium Hexametaphos Phate<br>Sodium Phosphate<br>Sodium Polyphosphates<br>Sodium Pyrophosphates<br>Sodium Triphosphate (To Retain Moisture)<br>Sodium Tripolyphophate<br>Tripotassium Phosphate<br>Trisodium Phosphate
**Sweetener***	Acesulphame Potassium<br>Allulose<br>Aspartame<br>Enzymatically Modified Stevia Glucosyl<br>Erythritol<br>Hydrogenated Starch Hydrolysate<br>Isomalt<br>Isomaltulose<br>Lactitol<br>Lactitol Monohydrate<br>Maltitol<br>Maltitol Syrup<br>Mannitol

	Monk Fruit & Associated Derivatives<br>Neotame<br>Polyglycitol Syrup<br>Rebaudioside-A (Stevia Leaf Extract)<br>Saccharins<br>Sorbitol<br>Sorbitol Syrup<br>Stevia Extract<br>Steviol Glycosides (Stevia)<br>Sucralose<br>Tagatose<br>Trehalose<br>Xylitol
**Thickener***	Acacia Extract<br>Acacia Or Gum Arabic<br>Acetylated Distarch Adipate (Maize)<br>Acetylated Distarch Phosphate (Potato)<br>Agar<br>Alginic Acid<br>Ammonium Alginate<br>Arrowroot Flour<br>Beta-Cyclodextrine<br>Beta-Glucan Concentrate<br>Calcium Alginate<br>Calcium Carrageenan<br>Calcium Caseinate<br>Calcium Stearate<br>Carbohydrate Gums<br>Carob Flour<br>Carrageenan<br>Chicle<br>Citrus Pectin<br>Corn Binder<br>Corn Fiber<br>Corn Products<br>Corn Starch<br>Dextrin<br>Dioctyl Sodium Sulphosuccinate<br>Distarch Phosphate<br>Ethyl Methyl Cellulose<br>Gellan Gum<br>Glucomannan (II)

Guar Gum
Gum Base
Hydroxypropyl Cellulose
Hydroxypropyl Distarch Phosphate
Karaya Gum
Konjac Flour
Konjac Or Konjac Gum
Locust Bean or Carob Bean Gum
Modified Starch (All Varieties)
Oat Bran
Oat Concentrate
Oat Powder
Pectin (440, 440A, 440B)
Polydextrose
Potato Dextrin
Potato Flour
Propylene Glycol Alginate
Psyllium Extract
Psyllium Fiber
Psyllium Husk Powder
Resistant Dextrin
Resistant Maltodextrin
Resistant Starch
Seaweed Extract
Seaweed Powder
Sodium Alginate
Soluble Corn Fiber
Soluble Fiber
Soluble Tapioca Fiber
Starch Sodium Octenyl Succinate
Sugar Beet Fiber
Sweet Potato Flour
Tamarind Seed Gum
Tapioca Dextrin
Tara Gum
Tragacanth Gum
Whole Algal Flour
Xanthan Gum
Yam Flour

Table S2. Ingredients classified as containing trans-fat.

**Ingredient**
Canola Oil
Cottonseed Oil
Diacetyltartaric & Fatty Acid Esters of Glycerol
Diglycerides
Distilled Monoglycerides
Esters Of Fatty Acids
Fatty Acids
Fractionated Palm Oil
Fully Hydrogenated Oils (all varieties)
Hydrogenated Oils (all varieties)
Hydrogenated Vegetable Shortening
Interesterified Oils (all varieties)
Lactic & Fatty Acid Esters of Glycerol
Lecithin
Margarine (Palm)
Mono And Diglycerides of Fatty Acids
Monoglycerides
Oil Blend
Palm Fat
Palm Oil
Partially Hydrogenated Oils (all varieties)
Rapeseed Oil
Shortening
Shortening Powder
Soy Bean Oil
Sucrose Esters of Fatty Acids
Vegetable Shortening (all varieties)

Table S3. Ingredient product count by aisle, category, and organic status.

Aisle	Category	Conventional (n =)	Organic (n=)
**Baby Food**	Baby Formula	5	1
	Cereal Bars & Cereal Snacks for Babies	1	17
	Cereal for Babies	27	14
	Cookies for Babies	4	11
	Crackers & Biscuits for Babies	6	14
	Fruit & Vegetable Snacks for Babies	11	29
	Juice & Drinks for Babies	23	11
	Meals for Babies	36	26
	Meat Sticks for Babies	1	0
	Other Baby Food	11	6
	Puffs for Babies	10	46
	Pureed Baby Food & Pouches	195	525
	Yogurt for Babies	13	7
	Total	343	707
**Bakery**	Brownies	186	1
	Carrot Muffins	3	0
	Cheese Muffins	1	0
	Chocolate Chip Muffins	43	0
	Chocolate Muffins	18	0
	Other Savory Muffins	17	0
	Other Sweet Muffins	143	0
	Pumpkin Muffins	10	0
	Zucchini Muffins	1	0
	Total	422	1
**Baking & Dessert Mixes**	Biscuit Dough	18	0
	Biscuit Mixes	25	1
	Bread, Roll & Muffin Mixes	83	2
	Brownie & Dessert Mixes	108	7
	Cake Mixes	191	5
	Cookie Doughs	32	0
	Cookie Mixes	127	5
	Cornbread Mix	24	0
	Dumpling & Savory Pancake Mixes	10	0
	Muffin Mix	63	1
	Other Baking Mixes	24	2
	Other Dessert Mixes	53	3
	Other Dough	6	0
	Other Frozen Bread	35	0
	Other Non-Dessert Baking Mixes	8	1

	Pastry Dough	14	0
	Pie Dough	13	0
	Pizza & Pie Crust Mixes	9	0
	Scone Mixes	3	0
	Total	846	27
**Baking Decorations**	Decorating Gels	6	0
	Food Coloring	5	0
	Frosting & Icing	119	3
	Other Dessert Toppings	78	0
	Total	208	3
**Baking Ingredients**	Baking Chocolate	36	7
	Chocolate Chips	96	20
	Cocoa	1	0
	Mincemeat Pie Filling	2	0
	Pastry & Pie Crust Doughs & Shells	4	0
	Pastry & Pie Fillings	129	8
	Corn Syrup	22	0
	Molasses	1	0
	Total	291	35
**BBQ & Other Sauces**	Asian Sauces	2	0
	Other Ethnic Sauces	210	22
	Peanut Sauce	12	2
	Sloppy Joe Sauce	29	0
	Sweet & Sour Sauce	21	0
	Teriyaki Sauce	82	6
	Worcestershire Sauces	32	4
	Total	388	34
**Breads, Bagels & Rolls**	Bagels	158	12
	Baguettes & French Breads	66	4
	Breadsticks	30	0
	Brioche	58	0
	Challah	4	0
	Ciabatta	27	2
	English Muffins	107	7
	Flatbreads	37	2
	Focaccia	3	0
	Hawaiian Rolls	14	0
	Italian Breads	59	2
	Multigrain Breads	62	22
	Naan	10	1
	Oatbran Breads	31	6
	Other Breads	234	34
	Other Wholegrain Bread	38	13

	Pita & Pocket Breads	30	5
	Potato Breads	24	0
	Pumpernickel	7	0
	Rye Bread	49	1
	Sourdough Breads	73	5
	Sweet Rolls	27	0
	Tortillas & Wraps	59	6
	Wheat Bread	233	15
	White Breads	227	25
	Pizza Dough	15	3
	Dinner Rolls	70	0
	Flax Breads & Buns	2	0
	Frozen Bread Loaves & Slices	22	0
	Frozen Bread Rolls	30	4
	Frozen Dough	0	1
	Frozen Garlic Bread	90	0
	Frozen Muffins, Biscuits & Bagels	47	0
	Garlic Bread	20	2
	Hamburger Buns	250	13
	Hot Dog Buns	176	8
	Raisin and Cinnamon Bread	46	2
	Spelt Bread	1	1
	Sprouted Breads & Buns	25	46
	Sweet Breads	20	0
	Taco Shells, Corn	40	4
	Taco Shells, Wheat	1	0
	Corn Tortillas	64	6
	Flour Tortillas	159	15
	Other Fruit Bread	31	0
	Total	2776	267
**Breakfast Foods**	Biscuit Sandwiches	14	0
	Breakfast Bars	56	0
	Breakfast Burritos & Wraps	4	0
	Breakfast Meals & Main Dishes	17	3
	Breakfast Meals & Main Dishes, Medium	4	0
	Breakfast Meals & Main Dishes, Small	36	3
	Croissant Sandwiches	2	0
	Flavored Crepes	8	0
	Flavored Pancakes	4	0
	Flavored Waffles	5	3
	Frozen Biscuit Sandwiches	62	0
	Frozen Breakfast Burritos & Wraps	59	3
	Frozen Breakfast Entrees & Meals	47	1

	Frozen Breakfast Main Dish (6 to 9.99 oz)	60	8
	Frozen Breakfast Meal (>= 10 oz)	7	0
	Frozen Breakfast Meals and Main Dishes (< 6 oz)	24	0
	Frozen Croissant Sandwiches	12	0
	Frozen Flavored Crepes	2	0
	Frozen Flavored French Toast	18	0
	Frozen Flavored Pancakes	55	2
	Frozen Flavored Waffles	140	8
	Frozen Meatless Breakfast Entrees	14	0
	Frozen Omelets	3	0
	Frozen Plain Crepes	1	0
	Frozen Plain French Toast	15	1
	Frozen Plain Pancakes	13	3
	Frozen Plain Waffles	81	10
	Hash Browns	51	0
	Omelets	5	0
	Other Breakfast Sandwiches	10	0
	Other Frozen Breakfast Sandwiches	83	0
	Pancake & Waffle Mixes	202	14
	Plain French Toast	2	0
	Plain Pancakes	4	0
	Plain Waffles	2	1
	Maple Syrup	8	12
	Total	1130	72
**Broths & Other Cooking Sauces**	Cooking Wines	28	0
	Hollandaise	1	0
	Other Cooking Sauces	145	4
	Red Clam Sauce	1	0
	Scampi Sauce	3	0
	Sherry	1	0
	Soy Sauce	100	24
	Stir Fry Sauce	40	2
	Vinegar	110	12
	White Clam Sauce	4	0
	Total	433	42
**Cakes & Pastries**	Apple Pie	49	0
	Blueberry Pies	10	0
	Cakes	537	0
	Carrot Cakes	18	0
	Cheesecake	123	2
	Cherry Pies	30	0
	Cinnamon Roll	32	0
	Coffee Cakes	34	0

	Creme Pies	11	0
	Croissants	46	0
	Cupcakes	282	0
	Danishes	76	0
	Donuts	326	1
	Eclairs	8	0
	Key Lime Pies	3	0
	Lemon Meringue Pies	6	0
	Marshmallow Rice Treats	114	2
	Other Cake Novelties	100	0
	Other Pastries	162	2
	Peach Pies	10	0
	Pecan Pies	20	0
	Pound Cake	39	0
	Pumpkin Pies	18	0
	Scones	19	0
	Sponge Cake	38	0
	Streusel	7	0
	Toaster Pastries	280	2
	Tres Leche Cake	6	0
	Turnover	2	0
	Upside Down Cake	2	0
	Angel Food Cake	7	0
	Frozen Cinnamon Rolls	13	0
	Total	2428	9
**Candy**	Assorted Candy Packs	27	1
	Assorted Chocolate	171	0
	Candied Apple	21	0
	Candy Bars	161	4
	Caramels, Toffee & Fudge	77	6
	Chewy Candy	568	22
	Chocolate Bars & Confection	1258	167
	Gumdrops	28	0
	Hard Candy	207	7
	Licorice & Twists	76	7
	Lollipops	129	4
	Marshmallow Candies	84	0
	Marshmallow Creme & Fluff	9	0
	Marshmallows	117	0
	Nougat & Marzipan	25	0
	Other Candy	312	10
	Other Candy Barks & Brittles	48	12
	Peanut Brittle	16	0

	Peanut Butter Flavored Candy, Cups & Bars	224	16
	Peppermint	131	5
	Promotional & Seasonal Candy	877	6
	Soft & Gummy Candy	644	18
	Sour & Tart Candy	95	0
	Sugar Free Candy	42	0
	Swedish Fish	47	2
	Taffy	80	0
	Toffee	31	0
	Truffles	181	30
	Total	5686	317
**Canned & Prepared Beans**	Baked Beans	114	7
	Black Beans	72	30
	Black-Eyed Beans	19	0
	Butter Beans	18	0
	Chili Beans	61	6
	Garbanzo Beans	45	23
	Great Northern Beans	14	7
	Kidney Beans & Red Beans	114	32
	Lima Beans	11	0
	Mixed Beans	6	10
	Navy Beans	16	2
	Other Beans	48	16
	Pinto Beans	71	21
	Refried Beans	107	24
	White Beans	20	1
	Total	736	179
**Canned & Prepared Fruit**	Apricots	17	0
	Berries	2	0
	Canned & Prepared Apples	8	1
	Cherries	20	0
	Cranberry Sauce	24	11
	Grapefruit	5	0
	Mandarins & Oranges	71	1
	Mixed Fruit Salad	112	1
	Other Fruit	24	6
	Parfait & Mixed Fruit Cups	76	3
	Peaches	162	14
	Pears	104	12
	Pineapple	152	8
	Total	777	57
**Canned & Prepared Tomatoes**	Chopped & Sliced Tomatoes	12	0
	Crushed Tomatoes	90	24

	Diced & Crushed Tomatoes	3	0
	Diced Tomatoes	407	70
	Stewed Tomatoes	75	1
	Tomato Paste	80	10
	Tomato Puree	26	8
	Tomato Sauce	148	22
	Tomato Sauce (Flavored)	19	1
	Whole & Peeled Tomatoes	109	19
	Total	969	155
**Canned & Prepared Vegetables**	Canned & Jarred Mixed Vegetables	50	0
	Canned & Jarred Mushrooms	90	14
	Canned Artichokes	65	1
	Canned Asparagus	25	0
	Canned Beets	67	4
	Canned Cabbage	7	0
	Canned Carrots	50	0
	Canned Corn	177	20
	Canned Green Beans	170	19
	Canned Green Chilies	38	4
	Canned Hearts of Palm	13	1
	Canned Okra	1	0
	Canned Onions	2	0
	Canned Peas	87	15
	Canned Peas & Carrots	16	0
	Canned Potatoes	38	0
	Canned Pumpkin	1	0
	Canned Sauerkraut	51	7
	Canned Spinach	10	0
	Canned Sweet Potatoes & Yams	14	1
	Other Canned & Jarred Vegetables	65	3
	Bruschetta	34	0
	Capers	19	8
	Jalapeno Peppers	95	9
	Olives	550	47
	Other Deli Goods	248	19
	Peppers	168	8
	Pickled Garlic	20	2
	Pickles	594	28
	Relish	135	10
	Sun Dried Tomatoes	27	6
	Tapenade & Other Vegetable Spreads	54	0
	Total	2981	226

**Canned Meals & Chili**	Canned Beans & Wieners	8	0
	Canned Beef Stew & Chili	17	0
	Canned Chicken Stew & Chili	12	0
	Canned Chili Sauce	20	0
	Canned Chili with Meat and Beans	66	0
	Canned Chili with Meat and No Beans	41	0
	Canned Chili Without Meat and With Beans	13	4
	Canned Pork & Beans	31	0
	Canned Ravioli	21	2
	Canned Turkey Stew & Chili	2	0
	Other Canned Chili & Stews	3	0
	Other Canned Meals	64	2
	Canned Hash	26	0
	Total	324	8
**Cheese & Cheese Alternatives**	Alternative/Non-Dairy Cheeses	119	32
	Block Cheese	729	55
	Canned Cheese	24	0
	Cheese Curds	36	0
	Cheese Cut for Crackers	71	0
	Cheese Dip	144	3
	Cheese in Rind (Not Wax)	22	0
	Cheese Sticks	80	6
	Cream Cheese	146	10
	Cubed/Chunked/Crumbled Cheese	141	12
	Flavored Cottage Cheese	58	0
	Flavored Cream Cheese	95	0
	Fondue	3	0
	Goat Cheese (including feta)	248	10
	Grated Cheese	109	3
	Individually packaged (not stick or string) Cheese	44	0
	Novelty Cheese Products Cheese	5	0
	Other Cheeses	42	0
	Plain Cottage Cheese	201	15
	Processed Cheese Block Cheese	52	3
	Shaved Cheese	19	0
	Shredded Cheese	610	35
	Shredded Mexican style Cheese	116	9
	Sliced & Singles Cheese	620	49
	Soft Cheese	164	3
	Spreadable Cheese	129	3
	String Cheese	123	12
	Total	4150	260
**Chips & Snacks**	Bagel Chips	9	1

Beef Sticks & Jerky	296	23
Cheese Puffs	317	22
Chickpea Snacks	67	9
Children's Snack Bars	49	69
Coated Pretzels	176	0
Corn Kernel Snacks	21	0
Cracker Crisps	68	7
Cracker Sticks	1	0
Flavored Popcorn (Popped)	482	41
Granola Bars	257	46
Lunchables	73	2
Meat Snacks	63	2
Meat Snacks (Vegetarian)	25	2
Onion Ring Snacks	27	3
Other Chips & Snacks	329	11
Other Lunch Snack Combinations	256	10
Other Meat Sticks & Jerky	174	0
Other Snacks	241	31
Pita Chips	63	3
Pork Rinds	169	0
Pork Sticks & Jerky	86	0
Potato Chips & Potato Snacks	1398	27
Pretzel Chips	16	0
Pretzel Crackers	4	1
Pretzels	255	9
Rice Cakes	55	33
Rice Chips	7	0
Rice Snacks	61	11
Salmon Sticks & Jerky	9	0
Seaweed	12	13
Sesame Sticks	25	0
Snack Mixes	258	10
Sweet Potato Chips	35	4
Tortilla & Corn Chips	592	93
Trail Mix Bars	35	0
Turkey Sticks & Jerky	57	7
Variety Snack Packs	5	0
Veggie Snacks	230	58
Venison or Deer Sticks & Jerky	4	0
Wheat & Multigrain Snacks	53	5
Soy Snacks, Chips & Nuts	6	0
Total	6366	553

**Cold Cereals & Granola**	Bran Cereals	79	7
	Corn Cereals	190	21
	Granola Cereals	399	122
	Multigrain Cereals	163	7
	Oat Cereal	177	21
	Other Cold Cereal	515	64
	Rice Cereals	122	6
	Sprouted Cereals	0	17
	Sprouted Granola	0	4
	Wheat Cereals	129	6
	Total	1774	275
**Condiments**	Aiolis	6	1
	Barbecue Sauces	424	36
	Cheese Sauces	35	0
	Chicken Wing Sauces	73	1
	Chili or Hot Sauces	267	10
	Chili Paste	2	1
	Chutney	11	0
	Curry Paste	19	1
	Curry Sauce	57	9
	Duck Sauce	6	0
	Fish Sauce	5	0
	Green Enchilada Sauce	26	3
	Green Mole Sauce	6	0
	Hoisin Sauce	11	3
	Horseradish Sauce	40	0
	Ketchup	187	40
	Kimchi	34	2
	Miso Paste	6	0
	Mustard	257	46
	Negro Mole Sauce	5	1
	Other Mexican Style Sauces	74	2
	Other Sauces	110	5
	Oyster Sauce	9	0
	Plum Sauce	4	0
	Poblano (Red) Mole Sauce	12	1
	Red Enchilada Sauce	41	7
	Sandwich Spreads & Other Spreads	26	2
	Sesame Sauce	9	0
	Steak Sauces	56	5
	Taco Sauce	54	8
	Tartar Sauce	31	3
	Wasabi	3	0

	Total	1906	187
**Cookies & Biscuits**	Almond & Nut Cookies	33	1
	Animal Crackers	39	21
	Assorted Cookies & Biscuits	29	0
	Biscotti	47	0
	Cakes & Cookie Bars	21	0
	Chocolate Chip Cookies	261	16
	Chocolate Covered Cookies	94	2
	Cookie Bars	28	0
	Cookie Sticks	3	0
	European Cookies & Biscuits	139	1
	Fruit Filled & Flavored Cookies & Biscuits	145	3
	Fudge Cookies & Biscuits	62	0
	Ginger & Gingerbread Cookies & Biscuits	95	3
	Graham Crackers	92	32
	Granola Cookies	32	4
	Kids & Promotional Cookies	46	5
	Lady Fingers	1	1
	Macaroons	23	2
	Meringues	6	2
	Oatmeal Cookies	116	5
	Other Cookies & Biscuits	396	12
	Peanut Butter Cookies & Biscuits	40	1
	Plain Cookies & Biscuits	24	0
	Sandwich Cookies	436	7
	Shortbread	84	0
	Sugar Free Cookies	34	0
	Sweet Biscuit Snacks	75	4
	Vanilla & Sugar Cookies	281	3
	Wafers	170	5
	Total	2852	130
**Crackers**	Assorted Crackers	2	0
	Cheese & Peanut Butter	245	24
	Crackerbread	44	19
	Crackers & Dip	74	1
	Kids & Promotional Crackers	2	2
	Matzo & Kichel Crackers	8	0
	Nut Crackers	10	0
	Other Crackers	248	40
	Other Flavored Crackers	63	3
	Other Wheat Based Crackers	166	18
	Oyster Crackers	17	0
	Rice Crackers	51	6

	Saltine Crackers	52	6
	Sandwich Crackers	108	4
	Sesame Crackers	19	2
	Toasted Crackers	16	1
	Total	1125	126
**Dessert Toppings**	Whipped Toppings	61	0
	Butterscotch Chips	6	0
	Butterscotch or Caramel	18	2
	Carob Chips	1	1
	Chocolate Syrup	39	4
	Cream, Flavored & Whipping Creams	94	10
	Dairy Toppings	50	0
	Fondant	17	0
	Fruit Syrup	49	7
	Hot Fudge	10	0
	Maraschino Cherries	53	0
	Other Dessert Chips	26	3
	Other Fruit Toppings	13	0
	Total	437	27
**Frozen Desserts**	Apple Pie	14	0
	Blueberry Pie	8	0
	Cherry Pie	11	0
	Chocolate Creme Pie	4	0
	Frozen Cakes	28	0
	Frozen Non-Dairy Desserts	326	42
	Frozen Pastries	61	0
	Fudge Bars	39	5
	Ice Pops & Juice Bars	268	28
	Italian Ice	37	0
	Key Lime Pie	7	0
	Lemon Meringue Pie	4	0
	Other Frozen Desserts	81	2
	Other Pies	130	0
	Peach Pie	5	0
	Pecan Pie	4	0
	Pumpkin Pie	9	0
	Soy Desserts	0	1
	Total	1036	78
**Frozen Meals, Side Dishes & Snacks**	Burger Dinners	1	0
	Burritos	116	14
	Chimichanga	17	0
	Enchiladas & Fajitas	41	1
	Flatbreads & Panini's	26	0

French Fries	164	8
Frozen Asian Appetizers	60	1
Frozen Beef Based Appetizers	23	0
Frozen Beef Based Meals, Large	42	3
Frozen Beef Based Meals, Medium	75	1
Frozen Beef Based Meals, Small	8	0
Frozen Blintzes	2	0
Frozen Cheese Sticks, Cheese Nuggets, & Cheese Snacks	72	1
Frozen Chicken Based Appetizers	28	0
Frozen Chicken Based Meals - Large	155	9
Frozen Chicken Based Meals - Medium	261	1
Frozen Chicken Based Meals - Small	68	0
Frozen Chicken Nuggets, Wings & Strips	281	21
Frozen Corndogs	37	0
Frozen Egg Rolls	27	0
Frozen Falafel	13	0
Frozen Meat Pies	72	1
Frozen Meatballs	26	2
Frozen Meatloaf & Meatball Meals	47	2
Frozen Mini Burger & Sliders	41	0
Frozen Other Frozen Appetizers & Hors D'oeuvres	57	3
Frozen Other Frozen Mexican Style Appetizers	59	0
Frozen Pasta Based Appetizers	16	0
Frozen Pasta Meals	727	18
Frozen Pierogies	27	0
Frozen Popcorn Chicken	8	0
Frozen Pork Based Appetizers	3	0
Frozen Pork Based Meals, Large	11	0
Frozen Pork Based Meals, Medium	17	0
Frozen Pork Based Meals, Small	4	0
Frozen Potato Skins	16	0
Frozen Prepared Tacos & Mini Tacos	21	1
Frozen Pretzel Sticks	33	1
Frozen Rice Based Meals	3	1
Frozen Sandwiches, Paninis & Pouches	257	0
Frozen Sausage Based Meals	8	0
Frozen Seafood Based Appetizers	75	0
Frozen Seafood Based Meals, Medium	33	0
Frozen Seafood Based Meals, Small	8	0
Frozen Soups	48	8
Frozen Stuffed Peppers & Jalapenos	24	0
Frozen Turkey Based Meals, Large	16	0

	Frozen Turkey Based Meals, Medium	14	0
	Frozen Turkey Based Meals, Small	2	0
	Frozen Vegetable Based Appetizers	48	4
	Frozen Vegetable Based Meals, Medium	96	10
	Frozen Vegetable Based Meals, Small	10	3
	Mixed Frozen Vegetables	2	1
	Onion Rings	25	0
	Other Frozen Meals	58	2
	Quesadillas	15	0
	Seafood Based Meals, Large	8	0
	Taquitos	46	0
	Vegetable Based Meals, Large	44	1
	Total	3542	118
**Frozen Pizzas**	Bread & Single Serve Frozen Pizza	103	2
	Frozen Calzones	21	0
	Frozen Cheese Pizza	175	7
	Frozen Chicken Pizza	54	1
	Frozen Deluxe & Supreme Pizza	94	0
	Frozen Other Meat Pizza	175	0
	Frozen Pepperoni Pizza	156	2
	Frozen Sausage Pizza	55	0
	Frozen Seafood Pizza	156	1
	Frozen Vegetable Pizza	117	12
	Other Frozen Pizzas	103	1
	Total	1209	26
**Fruit & Vegetable Juices & Drinks**	Acai Juice	0	1
	Almond Water	1	0
	Aloe Waters	5	3
	Apple Juice	128	44
	Apricot Juice	2	0
	Beet Juice	4	4
	Beverage Concentrates	26	12
	Blueberry Juice	1	0
	Carrot Juice	0	2
	Cherry Juice	43	3
	Chia Beverages	1	4
	Ciders	40	13
	Coconut Juice	3	0
	Coconut Water	59	19
	Cranberry Juice	84	14
	Frozen Fruit & Juice - Fruit Puree	3	3
	Fruit Smoothie	48	28
	Grape Juice	71	17

	Grapefruit Juice	29	0
	Guava Juice	13	0
	Lemon Juice	30	10
	Lemonade and Limeade	259	89
	Lime Juice	21	0
	Mango Juice	28	1
	Mixed Berry Juice	40	6
	Mixed Fruit & Vegetable Juice	165	122
	Mixed Fruit Juice	482	132
	Orange Juice	195	19
	Other Beverages	305	91
	Other Juice Flavors	35	25
	Papaya Juice	3	0
	Passion Fruit Juice	11	1
	Peach Juice	11	0
	Pear Juice	4	1
	Pineapple Juice	21	0
	Pomegranate Juice	11	4
	Probiotic Non-Dairy Drinks	35	131
	Prune Juice	9	2
	Raspberry Juice	2	0
	Sparkling Fruit Juice	99	26
	Strawberry Juice	4	0
	Tangerine Juice	1	0
	Tomato Juice	60	5
	Vegetable Juice	67	9
	Watermelon Juice	18	4
	Total	2477	845
**Gelatins, Puddings & Pectins**	Bread Pudding	2	0
	Custard	10	4
	Flan	10	0
	Flan Mix	5	0
	Fruit Gelatins	86	0
	Gelatin Mixes & Desserts	189	0
	Pudding	228	14
	Pudding Mix	138	4
	Total	668	22
**Half and Half & Creamers**	Creamers (Dry, Flavored)	14	0
	Creamers (Dry, Plain)	19	0
	Creamers (Liquid, Flavored)	205	4
	Creamers (Liquid, Plain)	24	4
	Fat Free Half and Half	21	0
	Half and Half	80	28

	Low Fat Half and Half	2	0
	Non-dairy Creamers (Dry)	55	0
	Non-dairy Creamers (Liquid)	41	4
	Total	461	40
**Ice Cream & Frozen Yogurt**	Frozen Yogurt	58	5
	Gelato	142	0
	Ice Cream Bars	395	11
	Ice Cream Cake	52	0
	Ice Cream Cones	117	6
	Ice Cream Sandwiches	212	18
	Ice Cream with Candy	189	25
	Ice Cream with Cookies	121	8
	Ice Cream with Multiple Types of Desserts, Candies, Nuts, and Other Treats	808	33
	Ice Cream with Nuts	79	1
	Ice Cream with Topping in Stripes	181	3
	Multiple Flavor Ice Cream	117	3
	Other Ice Cream Novelties	79	2
	Sherbet	65	0
	Smooth Ice Cream	407	41
	Sorbet	60	6
	Total	3082	162
**Iced Teas**	Decaffeinated Iced Teas	10	6
	Kombucha & Other Fermented Beverages	76	250
	Lemon Flavored Iced Teas	70	12
	Other Flavored Iced Teas	336	116
	Red Iced Teas	0	1
	Sweet Iced Teas	72	8
	Yerba Mate Drinks	11	23
	Total	575	416
**Jams & Jellies**	Apple Flavored Jams & Jellies	17	0
	Apricot Flavored Jams & Jellies	27	4
	Blackberry Flavored Jams & Jellies	32	2
	Blueberry Flavored Jams & Jellies	17	6
	Boysenberry Flavored Jams & Jellies	3	0
	Cherry Flavored Jams & Jellies	22	5
	Fig Flavored Jams & Jellies	14	4
	Grape Flavored Jams & Jellies	75	6
	Mixed Fruit Flavored Jams & Jellies	76	9
	Orange Flavored Jams & Jellies	26	5
	Other Flavored Jams & Jellies	101	3
	Peach Flavored Jams & Jellies	25	1
	Pineapple Flavored Jams & Jellies	4	0

	Plum Flavored Jams & Jellies	11	0
	Raspberry Flavored Jams & Jellies	54	12
	Strawberry Flavored Jams & Jellies	109	13
	Total	613	70
**Milk & Alternatives**	1% Fat Buttermilk	14	1
	1% Fat Cow's Milk	89	32
	2% Evaporated Milk	3	0
	2% Fat Buttermilk	6	0
	2% Fat Cow's Milk	124	74
	Almond Milk Flavored	91	29
	Almond Milk Plain	60	27
	Blended Non-Dairy Beverages	2	3
	Cashew Milk Flavored	5	1
	Cashew Milk Plain	5	0
	Coconut Milk, Flavored	7	11
	Coconut Milk, Plain	37	32
	Condensed Milk	43	3
	Eggnog	47	7
	Evaporated Whole Milk	29	2
	Fat Free & Skim Buttermilk	7	0
	Fat Free & Skim Cow's Milk	100	37
	Fat Free & Skim Evaporated Milk	13	0
	Flavored Cow's Milk	152	37
	Flax Milk Flavored	4	0
	Flax Milk Plain	2	0
	Goat Milk	6	0
	Hazelnut Milk Flavored	1	0
	Hazelnut Milk Plain	2	0
	Hemp Milk Flavored	7	0
	Hemp Milk Plain	1	0
	Lactose Free Milk	77	8
	Oat Milk Flavored	18	3
	Oat Milk Plain	38	8
	Other Dairy Beverage	15	0
	Other Plant Based Milks	55	13
	Powdered Low-Fat Milk	1	1
	Powdered Milk, Non-Fat	22	0
	Powdered Whole Milk	14	0
	Rice Milk Flavored	1	0
	Rice Milk Plain	1	2
	Shakes	5	0
	Soy Milk Flavored	18	16
	Soy Milk Plain	12	20

	Soy Milk Plain, Reduced Fat	1	0
	Whole Buttermilk	2	0
	Whole Cow's Milk	128	55
	Total	396	81
**Oatmeal & Hot Cereals**	Cream of Wheat Cereals, Flavored	21	0
	Cream of Wheat Cereals, Plain	15	1
	Grits, Flavored	7	0
	Grits, Plain	22	0
	Oatmeal with Fruit	7	4
	Oatmeal, Flavored	239	52
	Oatmeal, Plain	28	2
	Oats, Plain	3	2
	Other Hot Cereals	25	18
	Prepared Oatmeal	29	2
	Total	396	81
**Pasta & Noodles**	Sprouted Pasta	1	2
	Angel Hair	34	4
	Barley, Quinoa & Other Pasta & Noodles	62	39
	Chow Mein & Lo Mein	16	0
	Egg Noodles	114	3
	Elbows & Macaroni	90	3
	Farfalle	15	1
	Fettuccine	31	0
	Fresh Vegetable Based Noodles & Rice	9	1
	Gnocchi	32	1
	Instant Noodles	58	7
	Lasagne	47	1
	Linguine	39	3
	Non-Grain Pastas	32	25
	Oriental Noodles	39	13
	Other Pasta Styles	194	25
	Pasta Shapes & Alphabet	18	2
	Penne	77	7
	Rigatoni	40	3
	Rotini	60	3
	Shell Shaped Pasta	50	2
	Shells and Cheese	83	23
	Spaghetti	137	15
	Tortellini & Ravioli	214	25
	Vermicelli	8	0
	Total	1500	208
**Pasta & Pizza Sauces**	Alfredo & Cream Based Sauce	88	8
	Arrabbiata	25	5

	Cheese Flavored Pasta Sauce	57	6
	Chunky Pasta Sauce	44	2
	Marinara Sauce	124	25
	Meat Flavored Pasta Sauce	74	0
	Mushroom Flavored Pasta Sauce	53	4
	Other Pizza and Pasta Sauces	112	17
	Pesto	83	10
	Pizza Sauce	63	11
	Traditional Italian Pasta Sauce	236	48
	Vegetable Based Pasta Sauce	16	5
	Vodka Sauce	30	1
	Total	1005	142
**Peanut Butter & Other Nut Butters**	Almond Butters	51	5
	Cashew Butters	12	7
	Flavored Almond Butter	69	3
	Flavored Cashew Butter	7	0
	Flavored Hazelnut Butter	25	3
	Flavored Macadamia Butter	6	0
	Flavored Peanut Butter	53	5
	Flavored Seed Butter	0	6
	Hazelnut Butter	3	1
	Other Nut & Seed Butters & Spreads	25	16
	Peanut Butter	267	61
	Peanut Butter & Jelly Spreads	8	0
	Pistachio Butters	2	0
	Sunflower Seed Butters	5	12
	Walnut Butter	4	1
	Total	537	120
**Powdered Drinks**	Berry Flavored Powdered Drinks	21	0
	Cherry Flavored Powdered Drinks	21	0
	Grape Flavored Powdered Drinks	45	0
	Grapefruit Flavored Powdered Drinks	1	0
	Lemon & Lime Flavored Powdered Drinks	22	0
	Lemonade Flavored Powdered Drinks	111	0
	Mixed Fruit Flavored Powdered Drinks	130	0
	Orange & Mandarin Flavored Powdered Drinks	39	0
	Other Fruit Flavored Powdered Drinks	43	0
	Other Powdered Drinks	75	14
	Powdered Iced Teas & Tea Mixes	155	9
	Powdered Instant Cocoa & Flavored or Malted Milk	255	15
	Powdered Smoothie Mixes	20	10
	Powdered Spiced Cider	7	0
	Raspberry Flavored Powdered Drinks	11	0

	Strawberry Flavored Powdered Drinks	21	0
	Total	977	48
**Prepared Meals**	Dehydrated Asian Dinner	1	0
	Dehydrated Chicken Dinner	2	0
	Mac & Cheese	270	35
	Other Dehydrated Dinners	33	9
	Other Microwaveable Meals	80	5
	Pasta Dinners	170	1
	Ready Made Meals & Rice Mixes	244	30
	Total	800	80
**Processed & Deli Meats**	Beef & Pork Franks & Wieners	70	0
	Beef Deli Meats	45	4
	Beef Franks & Wieners	103	7
	Beef Pastrami Deli Meats	11	0
	Beef Sausages	39	3
	Bologna	54	0
	Bratwurst	90	2
	Breakfast Sausages	60	1
	Canadian Bacon	14	0
	Canned Ham	17	0
	Canned Luncheon Meat	25	0
	Canned Pate & Meat Spreads	16	1
	Canned Sausage	25	0
	Canned Turkey	3	0
	Capicola	3	0
	Chicken Deli Meats	59	5
	Chicken Franks & Wieners	80	22
	Chorizo	40	2
	Ham	314	6
	Head Cheese & Sousse	3	0
	Liverwurst	4	0
	Maple Sausages	18	0
	Meat and Cheese Combination	92	1
	Meat Spreads & Liverwurst	16	2
	Mixed Deli Meat	20	0
	Mortadella	1	0
	Other Cured Meats	13	0
	Other Deli Meats	8	0
	Other Franks & Wieners	87	0
	Other Sausages	62	3
	Pepperoni	76	1
	Polish Sausage	57	2
	Pork	23	0

	Pork Bacon	326	7
	Pork Sausages	281	3
	Prosciutto	30	0
	Salami	209	1
	Turkey Bacon	42	2
	Turkey Deli Meats	306	13
	Turkey Franks & Wieners	47	3
	Turkey Pastrami Deli Meats	4	0
	Total	2793	91
**Raisins & Other Fruit Snacks**	All Other Berries	26	7
	Apple Sauce	212	56
	Apple Sauce with Other Fruit	45	50
	Banana Snacks	33	12
	Coconut Snacks	18	7
	Cranberries	52	18
	Dates	6	0
	Dried Apples & Apple Chips	32	3
	Dried Apricots	31	1
	Dried Cherries	17	8
	Dried Pineapple	25	4
	Figs	20	1
	Fruit Bars	154	54
	Fruit Squeeze	34	60
	Kids Fruit Snacks	287	79
	Mango	36	5
	Mixed Fruit Snacks	36	20
	Other Fruit Snacks	51	11
	Prunes & Plums	34	0
	Raisins	37	9
	Total	1186	405
**Salad Dressings**	Asian Dressings	14	4
	Balsamic Dressing	86	28
	Blue Cheese Dressing	58	6
	Caesar Dressings	82	15
	Cheese Dressings	8	0
	Cole Slaw Dressings	17	1
	French Dressings	45	4
	Garlic Dressing	10	3
	Ginger Dressing	12	4
	Greek Dressings	24	2
	Italian Dressings	131	13
	Mustard Dressings	37	8
	Onion Dressings	8	0

	Other Salad Dressings	102	14
	Poppyseed Dressings	27	4
	Ranch Dressings	195	35
	Raspberry Dressings	26	4
	Russian Dressings	4	0
	Sesame Dressings	15	7
	Strawberry Dressings	2	0
	Tahini Dressings	7	5
	Thousand Island Dressings	43	6
	Vinaigrette Dressings	86	29
	Yogurt Salad Dressings	32	3
	Total	1071	195
**Salad Toppings**	Bacon Bits	51	0
	Croutons	96	11
	Other Salad Toppings	108	17
	Total	255	28
**Salsa & Dips**	Babaganoush	1	0
	Bean Dip	16	4
	Caramel & Chocolate Dips	40	1
	Fruit Dips	11	0
	Guacamole Dip	122	6
	Hummus	276	53
	Mixed Flavor Salsa	7	0
	Onion Dip	60	4
	Other Dips	101	10
	Plain Salsa	666	72
	Ranch Dip	59	5
	Seafood Cocktail Sauce	67	4
	Sour Cream Dip	15	0
	Spinach & Artichoke Dip	19	0
	Spinach Dip	20	1
	Tzatziki	18	0
	Vegetable Dip	12	0
	Total	1510	160
**Side Dishes**	Dehydrated Tabouli	2	0
	Mashed Potatoes	29	0
	Pasta Salad	14	0
	Polenta	1	4
	Potato Salads	104	0
	Potatoes Ready to Heat	14	0
	Prepared Gravy	85	8
	Prepared Sausage Gravy	9	0
	Total	258	12

**Sodas**	Birch Beer	8	0
	Cherry Flavored Soda	61	1
	Creme Soda	65	0
	Diet Cola	202	0
	Diet Soda	221	0
	Ginger Ale	152	1
	Grape Flavored Soda	45	0
	Lemon & Lime Soda	127	3
	Orange Flavored Soda	88	3
	Other Sodas	421	17
	Regular Cola	198	0
	Root Beer	89	0
	Total	1677	25
**Soups & Soup Mixes**	Bean & Lentil Condensed Soup	11	0
	Bean & Lentil Soup	10	13
	Beef Condensed Soup	12	0
	Beef Soup	34	0
	Broths & Stocks	303	145
	Canned Broccoli Soup	2	1
	Canned Chicken Noodle Soup	55	5
	Cheese Condensed Soup	2	0
	Cheese Soup	6	0
	Chicken Condensed Soup	60	1
	Chicken Gumbo Soup	8	0
	Chicken Soup	50	4
	Clam Chowder	37	0
	Corn Chowder	8	3
	Cream Of Condensed Soup	140	11
	Cream-based Soup	3	0
	Dehydrated Soup Mixes & Bases	211	8
	French Onion Soup	2	1
	Instant Soups	147	8
	Matzo Ball Mixes	5	0
	Meatball Soup	14	1
	Minestrone	15	4
	Miso Soup & Pastes	5	5
	Other Chowders	5	0
	Other Chowders Condensed Soup	2	0
	Other Condensed Soup	7	0
	Other Seafood Bisques	1	0
	Other Soups	21	2
	Other Vegetable Bisques	5	1
	Pasta & Noodles Condensed Soup	3	0

	Pasta & Noodles Soup	43	11
	Pea Condensed Soup	1	0
	Pea Soup	3	0
	Potato Soups	10	0
	Prepared Soups	324	99
	Sausage & Other Meat Soup	3	0
	Seafood Condensed Soup	1	0
	Seafood Soup	1	0
	Tomato Bisque	5	0
	Tomato Condensed Soup	33	2
	Tomato Soup	12	5
	Vegetable Condensed Soup	13	0
	Vegetable Soup	17	5
	Total	1650	335
**Sour Cream**	Fat Free Sour Cream	7	0
	Flavored Sour Cream	9	0
	Light Sour Cream	32	0
	Reduced Fat Sour Cream	2	1
	Sour Cream	106	11
	Sour Cream, Non-Dairy Imitation	6	2
	Total	162	14
**Stuffings**	Stuffing	29	1
	Stuffing Mixes	45	2
	Total	74	3
**Tofu & Meat Alternatives**	Frozen Tofu	0	1
	Frozen Vegetables - Spinach	4	1
	Frozen Vegetarian Bacon	3	0
	Frozen Vegetarian Breakfast Sausages	16	3
	Frozen Vegetarian Chicken Items	52	0
	Frozen Vegetarian Deli Meats	2	0
	Frozen Vegetarian Meatballs	15	0
	Frozen Vegetarian Sausages, Brats & Franks	7	0
	Frozen Vegetarian Seafood Items	7	0
	Imitation Crab	29	0
	Other Vegetarian Meat Options	70	6
	Soy & Veggie Patties and Burgers	118	23
	Tofu (All Types)	13	34
	Vegetarian Bacon	2	0
	Vegetarian Breakfast Sausages	12	0
	Vegetarian Burgers	25	4
	Vegetarian Chicken Items	5	0
	Vegetarian Deli Meats	18	0
	Vegetarian Meatballs	7	0

	Vegetarian Sausages, Brats & Franks	31	0
	Vegetarian Seafood Items	5	0
	Total	441	72
**Yogurt & Yogurt Drinks**	Classic Fat Free Yogurt	149	12
	Classic Low Fat Yogurt	255	21
	Classic Yogurt	174	72
	Greek Fat Free Yogurt	188	9
	Greek Low Fat Yogurt	101	4
	Greek Yogurts	93	30
	Non-Dairy Almond Yogurts	44	4
	Non-Dairy Coconut Yogurt	52	9
	Non-Dairy Soy Yogurts	6	0
	Other Non-Dairy Yogurt	49	13
	Other Yogurts	24	7
	Tube Yogurts	32	22
	Yogurt - Kefir	72	76
	Yogurt Drinks	77	5
	Yogurt Parfait	16	0
	Yogurt with Cereal	8	2
	Yogurt-based Smoothie	72	23
	Yogurts with Fruit	115	9
	Yogurts With Other Toppings	94	3
	Total	1621	321
